# Discordance of DNA Methylation Variance Between two Accessible Human Tissues

**DOI:** 10.1038/srep08257

**Published:** 2015-02-09

**Authors:** Ruiwei Jiang, Meaghan J. Jones, Edith Chen, Sarah M. Neumann, Hunter B. Fraser, Gregory E. Miller, Michael S. Kobor

**Affiliations:** 1 Centre for Molecular Medicine and Therapeutics, Child and Family Research Institute, Department of Medical Genetics, University of British Columbia, Vancouver, BC, V5Z 4H4, Canada; 2 Department of Psychology and Institute for Policy Research, Northwestern University, Evanston, IL 60208, United States; 3 Department of Biology, Stanford University, Stanford, CA 94305, United States; 4 Human Early Learning Partnership, School of Population and Public Health, University of British Columbia, Vancouver, BC, V6T 1Z8, Canada

## Abstract

Population epigenetic studies have been seeking to identify differences in DNA methylation between specific exposures, demographic factors, or diseases in accessible tissues, but relatively little is known about how inter-individual variability differs between these tissues. This study presents an analysis of DNA methylation differences between matched peripheral blood mononuclear cells (PMBCs) and buccal epithelial cells (BECs), the two most accessible tissues for population studies, in 998 promoter-located CpG sites. Specifically we compared probe-wise DNA methylation variance, and how this variance related to demographic factors across the two tissues. PBMCs had overall higher DNA methylation than BECs, and the two tissues tended to differ most at genomic regions of low CpG density. Furthermore, although both tissues showed appreciable probe-wise variability, the specific regions and magnitude of variability differed strongly between tissues. Lastly, through exploratory association analysis, we found indication of differential association of BEC and PBMC with demographic variables. The work presented here offers insight into variability of DNA methylation between individuals and across tissues and helps guide decisions on the suitability of buccal epithelial or peripheral mononuclear cells for the biological questions explored by epigenetic studies in human populations.

Epigenetic analysis is gaining momentum in modern biomedical research, in part because of its potential to contribute to understanding complex phenotypes^1^,^2^. Among the many factors constituting the epigenome, the best studied is DNA methylation (DNAm), which primarily refers to a methyl group covalently attached to a cytosine in the context of CpG dinucleotides^1^,^2^,^3^. Rapid technological advancements allowing routine quantitative measurements of DNAm at multiple CpGs across a large number of subjects have facilitated the integration of DNAm in human population studies^4^,^5^. Epigenome-wide association studies (EWAS) are the cousin of genome-wide association studies (GWAS), primarily aimed at identifying DNAm differences associated with disease phenotype, progression, or predisposition^2^,^6^,^7^,^8^,^9^. These studies show that epigenetics is emerging as an important aspect of human health and disease due to its variability and potential for mediating the interaction between environmental associations and phenotypic outcomes^1^,^2^,^10^,^11^,^12^.

Population epigenetic studies face the challenge of integrating epigenetic variation with tissue specificity. Tissue specificity is perhaps the best understood fundamental property of epigenetic biology, with different cell types and tissues acquiring distinct epigenomes during development^13^,^14^. In contrast to specificity, variation of the epigenome between individuals is not as widely explored, yet it is of great importance for population epigenetics, as it is a prerequisite for the discovery of any epigenetic association with either exposure or phenotype. It is reassuring that several studies have recently documented the existence of inter-individual DNAm differences within a given tissue^15^,^16^,^17^,^18^. Recently, the relevance of DNAm variability in particular to disease models has been demonstrated. One study showed that DNAm variability of uterine cervix cells differed between subjects who developed non-invasive cervical neoplasia versus those who were free of the disease^19^. Another study examined the relationship between DNAm variability in peripheral blood cells and obesity, and showed that at certain sites, DNAm exhibited higher variability in case than controls, and these sites enriched for genes associated with obesity and obesity related diseases^20^. Finally, a study examining discordance for depression among monozygotic twins found differences in variance in affected versus unaffected twins^21^. Thus it appears that DNAm variability might be associated with disease risk and progression. Besides disease related variability, factors such as ethnicity, aging, environmental exposures, and genetic allelic variation together contribute to epigenetic variation between individuals^1^,^2^,^12^,^22^,^23^.

In part, tissue-specific DNAm and inter-individual variance is linked to distinct promoter structures and their epigenetic properties, particularly the presence of CpG islands (CGIs). CGIs are regions that harbor a higher than average density of CpG dinucleotides, which are otherwise underrepresented in the genome^1^,^2^,^3^,^24^. Approximately 70% of human gene promoters are associated with CpG islands, and methylation of CpG islands has been shown to be highly correlated with gene expression levels^4^,^5^,^24^,^25^. While several nuanced classifications for CGIs are in use, the one that provides the best enrichment discrimination separates CGIs into high CpG density islands (HCs), intermediate CpG density islands (ICs) and low CpG density islands (LCs)^2^,^6^,^7^,^8^,^9^,^24^,^26^. In somatic cells, DNAm patterns tend to be correlated with CpG density, as on average, HC regions have low levels of CpG methylation, while IC and LC regions have increasingly higher levels^1^,^2^,^10^,^12^,^18^,^26^,^27^,^28^,^29^. Epigenetic differences between tissues are primarily associated with DNAm differences in IC promoter regions, while reports are inconsistent as to which class is enriched for CpGs that vary between individuals^13^,^14^,^16^,^18^,^30^,^31^,^32^.

Currently, the vast majority of studies in the growing field of epigenetic epidemiology naturally rely on a limited number of easily accessible tissues such as blood and BEC due to their availability and non-invasive nature^10^,^15^,^16^,^17^,^18^,^33^. The former often are further processed to obtain peripheral blood mononuclear cells (PBMC), which are an immunologically relevant fraction of lymphocytes that lacks multinucleated granulocytes. Importantly, PBMCs are derived from mesoderm whereas BEC are derived from ectoderm, thus representing two different germ layers and distinct developmental origins.

Given the widespread use of either PBMCs or BECs for inferring population-level phenotypes, it is important to ask how these tissues compare in terms of variance, and to reconcile their DNAm variability with tissue specificity. Here, we compared matched PBMCs and BECs (obtained using cheek swabs) from a small community cohort of 25 healthy subjects at 998 CpG sites to examine how inter-individual variability differed across tissues. While we found that differences between tissues exceed differences between individuals in the same tissue, the latter variation had a strong tissue specific component. These tissue-specific differences were also reflected in exploratory association of DNAm with demographic variables. Collectively, these findings reveal important considerations for the selection of surrogate tissues for EWAS^10^,^19^,^33^.

## Results

We recruited 25 healthy adults from the Greater Vancouver area to our study cohort, and collected BEC and PBMC from each individual. This design allowed for a rigorous matched comparison between the two tissues, eliminating potential confounds due to inter-individual differences in environments or genetic background. DNAm of 1,506 promoter-associated CpGs biased towards cancer genes was quantitatively determined using an array-based approach^14^,^20^,^34^. After removing CpGs located on the sex chromosomes to avoid biases due to our cohort consisting of males and females and CpGs overlapping with SNPs or annealing to multiple regions in the genome, as well as 28 CpG sites where more than 3 samples had bad detection p-values, our final dataset consisted of 998 CpGs^14^,^21^.

### PBMCs and BECs had substantially different DNAm profiles

We first interrogated broad tissue differences in DNAm across the population. Each of the 25 individuals had higher mean PBMC DNAm than BEC DNAm, and this cross-tissue difference was statistically significant by a paired Wilcoxon ranked sum test p-value of 3.16e-14 (n = 25, two tailed) (Fig. S1A). While both tissues had the expected bimodal distribution of DNAm for each single CpG site across all subjects, there were noticeable differences in the specific fractions. Categorizing mean CpG methylation according to a published categorization schema, we found 62.4% of sites in BEC and 56.9% of sites in PBMC were hypomethylated (<20%), 16.7% in BEC and 20.3% in PBMC were heterogeneously methylated (20–80%), and 20.8% in BEC and 22.7% in PBMC were hypermethylated (>80%) (Fig. S1B)^35^.

**Figure 1:**
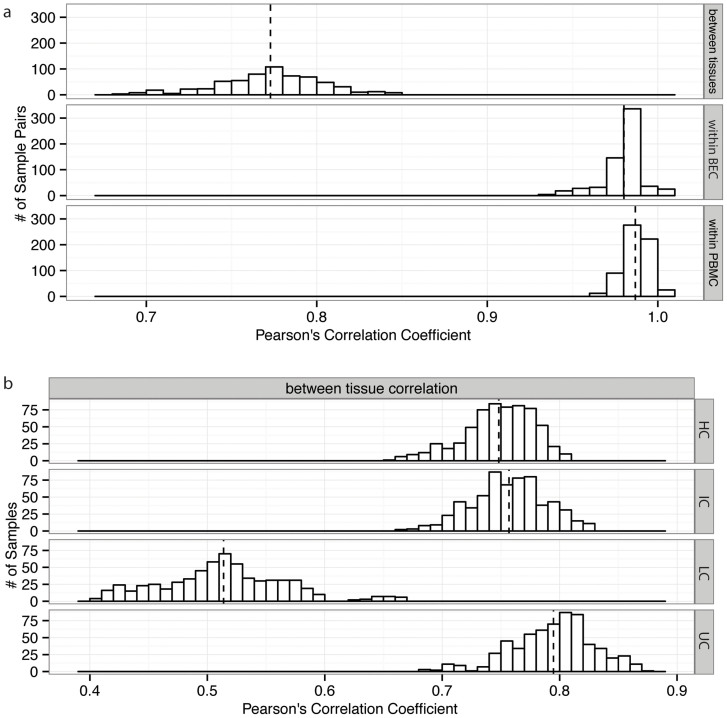
Correlation of DNA methylation was higher within than between tissues. (a) Distribution of Pearson's correlation coefficient for correlations within BEC, within PBMC, and between BEC and PBMC. In each instance, dashed lines represent the average correlation coefficient: within BEC = 0.980, within PBMC = 0.987, between tissue = 0.773. (b) Distribution of Pearson's correlation coefficient for BEC versus PBMC sample-wise correlation with CpG sites separated into four categories: low, intermediate, and high CpG density, and uncategorized. Low CpG density islands displayed the lowest correlation coefficients.

**Figure 2:**
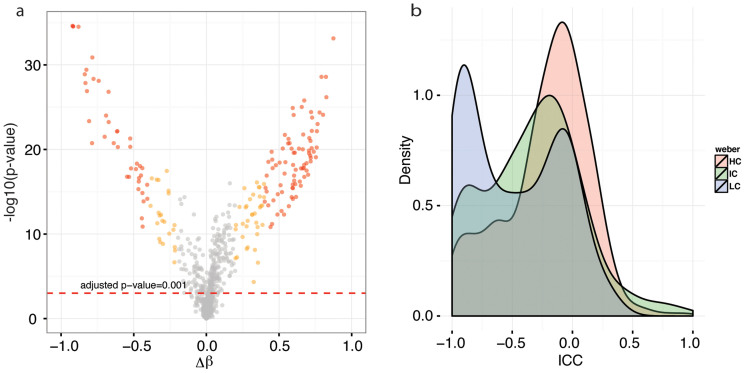
Tissue specific DNA methylation was enriched in regions of low CpG density. (a) Probewise tissue specific DNA methylation was evaluated by t-test p-value and Δβ. Categories of differentially methylated probes were identified by color. 12.1% of all CpG loci (red) had p-value < 0.001 and Δβ > 40%, and 17.8% of CpG loci (yellow) had p-value < 0.001 and Δβ > 20%; (b) Distribution of intra-class correlation coefficient (ICC) for high, intermediate, and low CpG density regions. Probes residing in regions of low CpG density had higher proportion of ICC values near −1.0.

To determine whether differences in DNAm between individuals or between tissues contribute more to probe-wise variability, we next compared between-individual and between-tissue correlations. Across the 998 CpG sites, the average two-tailed Pearson's correlation coefficient of DNAm profiles among individuals was 0.980 for BEC and 0.987 for PBMC, and the coefficient between the two tissues was 0.773 (Fig. 1A). Separating all probes into 212 LCs, 136 ICs, 503 HCs, and 147 uncategorized sites, we found that the relatively low between-tissue correlation was driven by LCs sites, which had a mean correlation of 0.514 between PBMCs and BEC; on the other hand, HC and IC had mean correlations of 0.748 and 0.757, respectively, and uncategorized sites had a higher correlation of 0.795 (Fig. 1B). To explore tissue-specific DNAm in more detail, we identified probes that were differentially methylated between BEC and PBMC using a paired two sample t-test (n = 25, two tailed) followed by Benjamini Hochberg (BH) false discovery rate correction^36^. With this approach, we found that 537 CpGs (53.8%) were significantly different between PBMC and BEC with p-value < 0.001 (Fig. 2A). Of these significant sites, 178 (17.8%) had a difference in average beta value (Δβ) > 0.2 across the two tissues, and 121 of the 178 (12.1%) had Δβ > 0.4 (Fig. 2A). Since the collection of 178 probes passed statistical threshold for significance, and was of reasonable size, we conducted functional enrichment analysis on the 178 highly tissue specific probes (located in 137 unique genes) using the online bioinformatics tool DAVID to determine if differentially methylated sites were representative of cellular processes specific to BEC or PBMCs^26^,^37^. Using the recommended cutoff of enrichment score ≥1.3, two clusters were considerably enriched (Table S2). Cluster 1 contained biological process (BP) GO term immune system process (GO: 0002376) accounting for 30 genes, as well as molecular function GO term cytokine activity (GO: 0005125) accounting for 11 genes. Cluster 2 contained cellular component GO term extracellular region (GO: 0005576) accounting for 48 genes. Both cluster 1 and cluster 2 seemed to be enriching for PBMC and BEC specific processes, respectively, suggesting a reassuring level of association between DNAm and the unique functionalities of different cell types.

Stratifying tissue-specific DNAm loci according to CGI density, we found that LC regions, which constituted only 21% of all CpG sites on the array (212 out of 998 probes were located in LC regions), accounted for 31% of the 449 differentially methylated sites (hypergeometric p-value of 1.02e-12 indicating significant enrichment), consistent with this class having the lowest correlation between BEC and PBMCs. To further corroborate this finding, we calculated probewise intraclass correlation coefficient (ICC) for each of the three LC, IC, and HC categories. ICC is a reliability index which, in this case, was used to measure the degree of between-tissue methylation difference: ICC values closer to −1 indicate larger difference between tissues than within tissues, while ICC values closer to +1 indicate larger within-tissue difference. Distribution of ICC values showed that the majority of HC and IC probes were clustered around ICC of 0, indicating equitable within and between tissues differences (Fig. 2B). On the other hand, LC probes were clustered around ICCs of 0 and −1, with the latter displaying the most prominent peak, which means that the majority of LC probes harbored larger between than within tissue differences. These results thus suggested that tissue specific DNAm were enriched for regions of low CpG density.

### Variability of DNAm was largely tissue-specific

**Figure 3:**
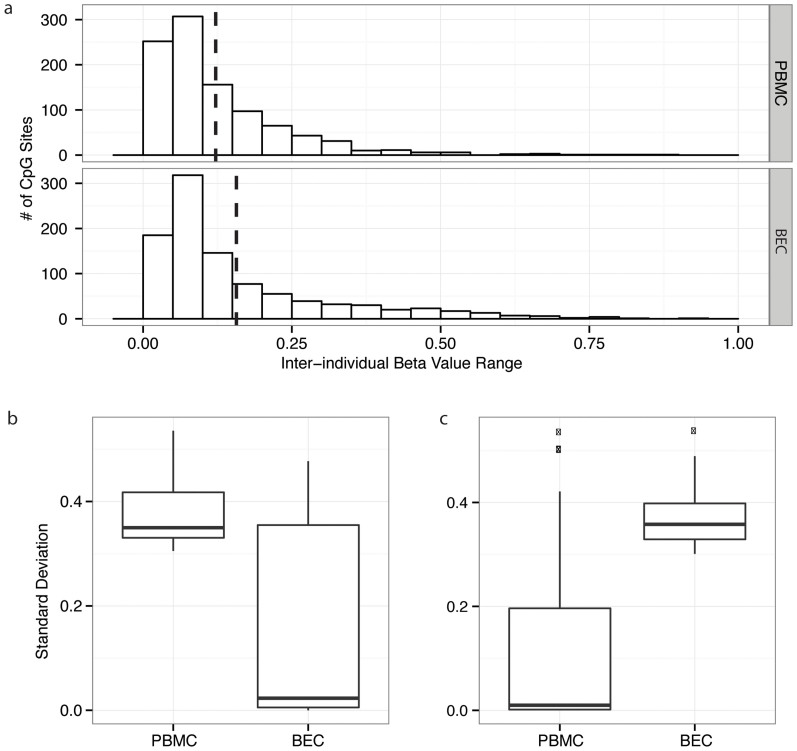
Variability in DNA methylation was tissue-specific. (a) Distribution of DNA methylation range of 998 CpG sites for the two tissues. BEC had larger probe methylation range than PBMC. (b) Boxplot of SD for 26 sites with SD > 0.3 in PBMC and the equivalent sites in BEC (c) Boxplot of SD for 55 sites with SD > 0.3 in BEC and the equivalent sites in PBMC. High SD in one tissue did not predict high SD in the other tissue.

Having defined tissue-specific DNAm, we next turned to inter-individual variability within each tissue and, in particular, how this variability compared between the two tissues examined. For each CpG site, we calculated the inter-individual range of beta values for both PBMC and BEC, and then visualized their overall distribution (Fig. 3A). BEC had a larger overall DNAm range than PBMC, which was confirmed statistically by ANOVA on the BEC and PBMC distributions (p-value of 2.05e-8). Next we determined the degree of variation for each CpG by calculating the standard deviation (SD) of each CpG site for the two tissues. In the combined BEC and PBMC data, we compared the number of highly variable probes across tissues, using SD > 0.1 and SD > 0.3 as two levels of cutoffs for high variability. Using a cutoff of 0.1, 123 (12.3%) of PBMC and 150 (15.0%) of BEC loci were variable; using a SD cutoff of 0.3, 26 (2.6%) of PBMC and 55 (5.5%) of BEC loci were variable (Fig. 3B). To determine the regions at which variable loci were most likely to be found, we performed hypergeometric tests on CpG density compositions, and found that variable loci (150 with SD > 0.1) in BEC were enriched for LC regions (p-value of 2.39e-5, 51 LC loci) and IC regions (p-value of 2.19e-2, 28 IC loci), but depleted of HC regions (p-value of 3.51e-8, 45 HC loci). Highly variable BEC loci with SD > 0.3 (55 loci) were enriched for LC regions (p-value of 0.01, 18 LC loci), but depleted of HC regions (p-value of 0.02, 20 HC loci). Similar trends were observed for variable loci in PBMCs (SD > 0.1, 123 loci), which were enriched for LC regions (p-value of 1.57e-2, 35 LC loci) and IC regions (p-value of 9.38e-3, 25 IC loci), but depleted of HC regions (p-value of 4.59e-3, 48 HC loci), though highly variable PBMC loci (SD > 0.3, 26 loci) were not enriched or depleted of any particular region.

**Figure 4:**
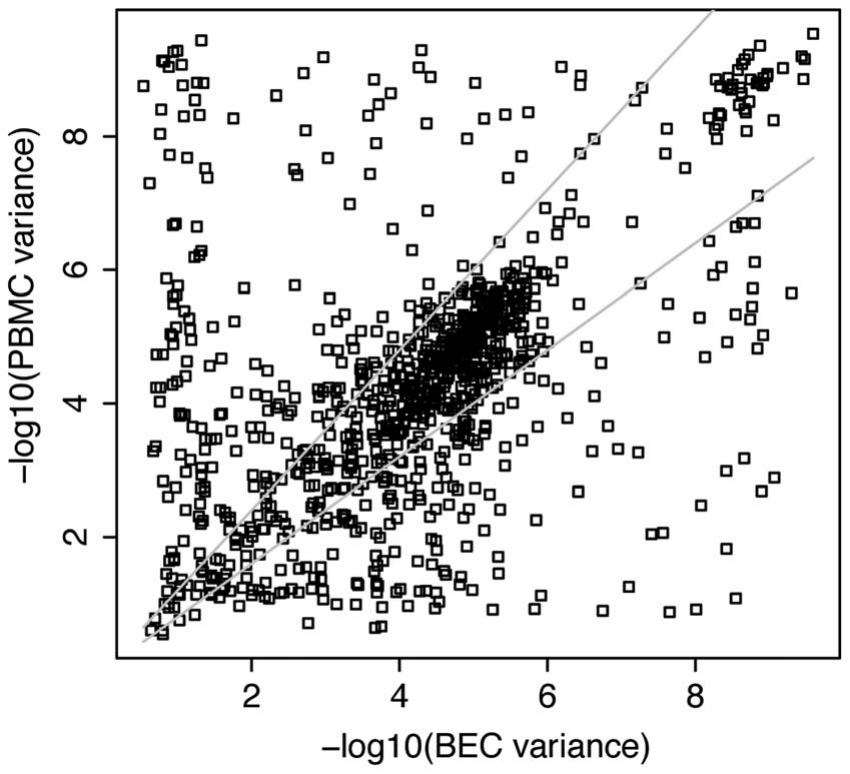
DNA methylation variability was tissue specific. 584 probes had PBMC variance that deviated <±20% from their corresponding BEC variance. 414 probes were found beyond the ±20% threshold, further demonstrating the variance discordance between BEC and PBMC.

Taking advantage of our matched study design, we compared variability of sites between BEC and PBMC directly by calculating the variance for each probe, and determining equality of variance using Levene's test^38^. This site-by-site evaluation of variance equality showed that 346 (34.7%) loci did not have equitable variance across the two tissues (Levene test p-value <0.05). Of these loci, 144 showed larger BEC variance, and 202 showed larger PBMC variance. This is in contrast to the earlier analysis where we found more sites with large SD in BEC when examining each tissue separately. Plotting the variance of PBMC versus the variance of BEC, we obtained a Pearson's correlation coefficient of 0.26 (Fig. 4). By setting variance in BEC as a reference, we searched for probes for which the PBMC variance deviates more than ±20% from the BEC variance. Using this method we found 584 probes residing within the ±20% interval, and 414 probes outside the interval (Fig. 4). These observations indicated that variance was specific to the tissue; a probe that was highly variable in BEC would most likely not behave the same way in PBMC.

We next sought to find out how similar between-individual patterns of DNA methylation were across tissues. In other words, if individual *a* had higher DNAm than individual *b* at probe *i* in PBMC, would the same or opposite trend be observed in BEC. We carried out a probewise Pearson's correlation of the DNAm values between BEC and PBMC and found that only 29 probes displayed relatively good correlation between the two tissues (absolute Pearson's correlation coefficient >0.5) (Fig. S2). A total of 23 probes out of 29 survived false discovery rate (FDR) correction with a medium confidence FDR of 0.25, and 13 probes survived using a high confidence FDR of 0.05. This indicated that in most cases, the relative individual level of DNAm at a specific probe was not translatable across tissues.

### Tissue-specific association between DNAm and demographic factors

**Figure 5:**
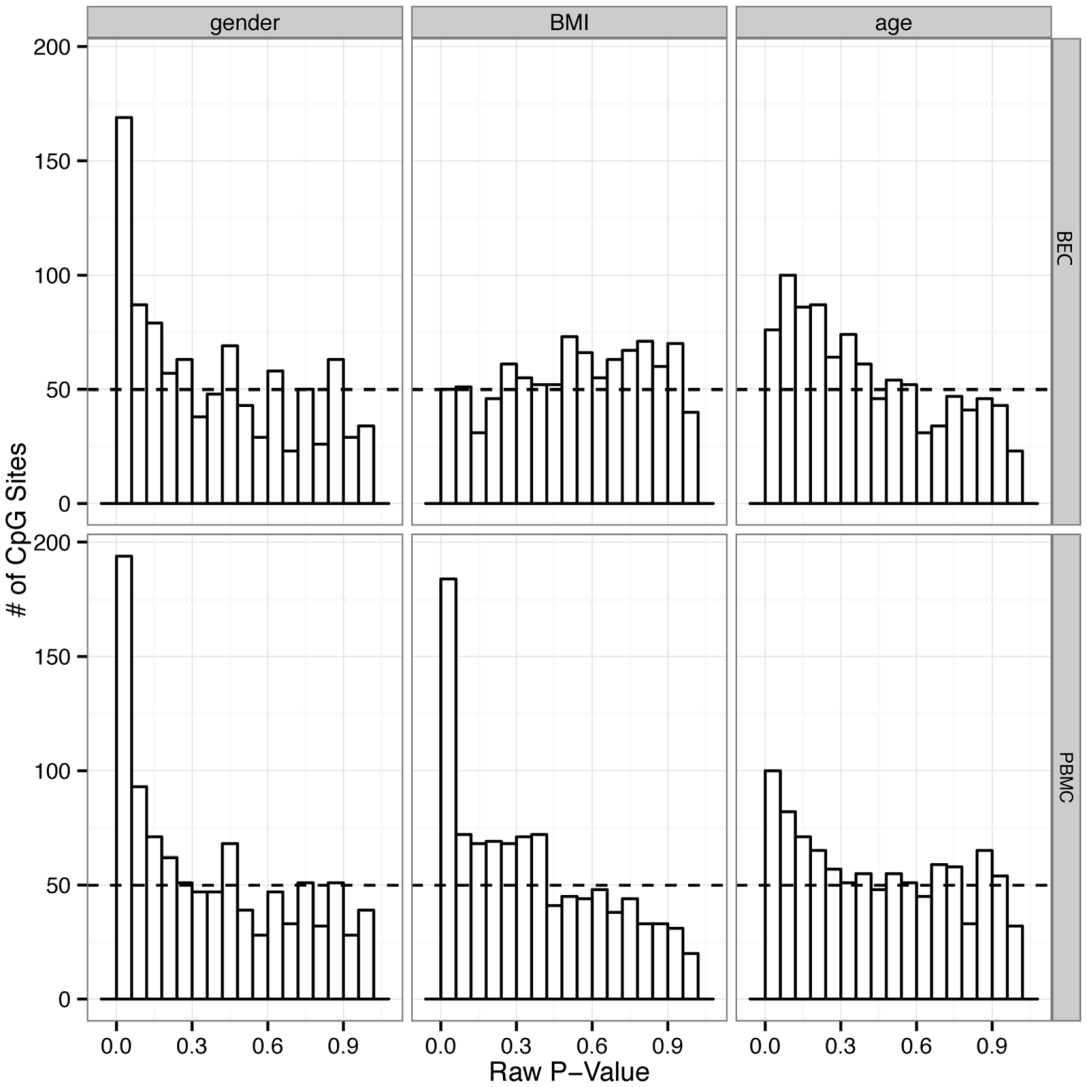
Demographics factors were associated with DNA methylation changes in both PBMC and BEC. Distribution of unadjusted p-values for association between gender (left), BMI (middle), and age (right) in BEC (top) and PBMC (bottom). Left-skewed p-value distributions showing an enrichment of CpG sites with small p-values suggested correlations of certain demographic factors with DNA methylation. This is in contrast to the relatively uniform p-value distribution of BMI and BEC, suggesting lack of such correlation.

We next performed an exploratory analysis to examine each tissue individually for associations with a small set of demographic factors that we had collected in our cohort. Given the small size of our cohort, these analyses explore how the differences in variability between BEC and PMBC affect their association with demographic factors, rather than constituting a statistically significant determination of specific probes that are associated with these demographics. As a rough guide to the relative associations between DNAm and the demographics in our tissues, in addition to the number of associated probes we also examined the distributions of unadjusted p-values. As we have previously described, left skewing of p-value distributions is indicative of a higher likelihood of association^18^. The variables examined included: age (26–45), gender (15 male versus 10 female), ethnicity (18 Caucasian versus 7 non-Caucasian), and body mass index (BMI, 18.18–47.95). We used a two tailed Wilcoxon rank sum test for gender and ethnicity, and a Pearson's two-sided correlation for age and BMI, and false discovery rate (FDR) was corrected with Storey's qvalue method^39^. No specific probes were found to be associated with age, but skewed p-value distributions indicated a signature of age (Fig. 5). Using a FDR ≤ 0.05 (high confidence), in BEC 0 probes were associated with gender while 4 probes were associated with BMI. The p-value distributions for the association of gender and BMI with methylation in BEC were quite different (Fig 5). Gender showed the left-skewing associated with higher confidence associations, while BMI showed the flat distribution that generally indicates low likelihood of association (Fig. 5). In PBMCs 40 probes were associated with gender (10 with Δβ > 5%), and 51 probes were associated with BMI (31 with Δβ > 5% between subjects with highest and lowest BMI) using an FDR of 0.05 (Table S3). These results were both supported by their skewed p-value distributions (Fig. 5).

## Discussion

In this study, we took advantage of matched tissue samples from a cohort of healthy adults to examine basic distribution of DNAm, its variation between individuals, and its broad association with selected demographic factors for 998 CpG methylation sites. Given the fundamental role of the epigenome in specifying tissue differences, it was not surprising that a large fraction of CpGs had substantially different DNAm levels between the two tissues. While the epigenetic differences between tissues greatly overshadowed inter-individual CpG methylation differences in the same tissues, the latter nevertheless encompassed a number of loci and revealed some important biological principles. Specifically, we showed that variation in DNAm can be tissue specific, and in addition, exploratory analysis showed that the association of DNAm with demographic variables was at least partly tissue-specific as well. As such, these results have practical implications for sample selection for population epigenetics studies.

With regards to tissue-specific DNAm marks, our results derived from matched samples largely confirmed principles from a large body of published data, most of which were derived from unmatched samples. We discovered that CpGs differing between tissues were not only significant in number but also large in the magnitude of absolute methylation difference. Reassuringly and consistent with the published literature, these CpGs resided primarily in LC regions, and consequently, these also had by far the lowest pairwise correlation across tissues^13^,^14^,^16^,^18^,^30^,^31^,^32^. This implies that DNAm of PBMC and BEC is more different at regions of low CpG density than at medium or high density. We also replicated the finding that BECs have overall lower DNAm levels than PBMCs^2^,^6^,^40^. The issue of genetic variants that affect DNAm, known as methylation quantitative trait loci or mQTLs, is a concern in many epigenetic studies^16^,^41^,^42^. Here, however, our matched design means that our comparisons across tissues are not confounded with respect to mQTLs, since we performed paired testing in samples from the same individual. When comparing across individuals, it is possible that mQTLs contribute to the variance in probes, however we believe this to be highly unlikely as the overall known percentage of CpG sites associated with mQTLs is very small, generally estimated between 1% and 7%^41^,^43^,^44^.

Inter-individual variability across human tissues has been investigated in several recent studies^15^,^16^,^17^,^18^. Our matched sample design allowed us to investigate inter-individual variability across the two most accessible human tissues. Highly variable probes were identified largely in low CpG density regions in both tissues, a finding which is consistent with published reports, though overall, studies are divided on which class of CpG island is the most variable^18^,^30^,^31^. In our study, several important epigenetic features differed substantially between the two tissues. First, we found that when examining each tissue separately, BEC had a larger variable range and contained more highly variable sites than PBMCs. Second, BEC had a stronger association between probe variability and CpG island type compared to PBMC. Third, and perhaps most importantly, CpGs that had a variable range in one tissue did not necessarily have the same range in the other tissue. In fact, variance of DNAm for a given site differed at over a third (34.7%) of loci tested. Also, despite having fewer sites with high variance overall, PBMCs had more sites with higher variability within the loci that showed discordant methylation between the two tissues. This observation was particularly important for epigenetic population studies that rely on these easily accessible tissues for indicators of DNAm variability; depending on which tissue is chosen for the study, the outcome of identified differentially variable CpG sites could be vastly different. This could then affect the tissue's associations with the biological and deterministic factors that are often of interest in epigenetics studies.

Consistent with the differences in variability across tissues, we next showed that associations between DNAm and a small set of demographic factors were also different between the two tissues. In particular, while both tissues had CpG sites that were associated with gender, only PBMCs demonstrated a substantial number probes that were statistically significant. Similarly, we identified more CpGs associated with BMI in PBMCs than in BEC, consistent with a recent report which also showed DNAm associations with BMI in PBMCs^45^. Although we did find an age signature from the positive skewing of p-value for both tissues, we did not find significantly related CpG sites. The lack of significant age-related sites could be due to the limited age range (26–45) of this study compared to studies dedicated to examining age-related changes in DNAm (generally <25 to >75), or to the relatively restricted distribution of the CpGs analyzed^46^,^47^,^48^,^49^.

Collectively, these results present somewhat of an interesting conundrum: BEC DNAm was more variable across individuals, yet PBMC DNAm was more strongly associated with the demographic variables tested here. In contrast, published work suggests that BECs are a more suitable tissue than peripheral blood for population epigenetic studies^40^. This was largely based on the finding that BECs containing more hypomethylated regions than PBMCs, which not only seem to overlap with hypomethylated regions in other tissues but also tend to cluster around disease associated SNPs^40^. However, given that PBMCs represent a circulating tissue directly involved in the immune and inflammatory systems and are thus functionally related to many major diseases, it is our opinion that in most cases, PBMCs would be the better choice^2^,^35^,^50^. It is of course possible that for specific studies, selection of the appropriate tissue might be context-dependent, but our results show that for population epigenetics studies more broadly, the implications of tissue-specific variability need to be carefully considered when assessing DNAm, in particular when the primary tissue of interest is not accessible.

## Methods

### Sample Preparation and DNAm Analysis

All experimental protocols were approved by the University of British Columbia's Research Ethics Board, and all methods were carried out in accordance with the approved guidelines. All subjects gave written consent before participating. Matching buccal and blood samples were collected from 25 adults in the Greater Vancouver Area. The PBMC fraction was extracted from blood using density gradient centrifugation, and for both BEC and PBMC, genomic DNA was extracted using standard techniques. To measure DNAm, 750ng of DNA was treated with the EZ DNAm kit (Zymo Research, Orange, CA, US) to convert unmethylated cytosines to uracil, after which DNAm at 1,536 CpG loci and 371 genes was evaluated using the Illumina GoldenGate Assay for DNAm profiling as described^34^. Briefly, for each CpG site there were two allele-specific probes annealing to either the methylated or the unmethylated sequence. Annealed probes are then paired to locus-specific oligos, after which the ligated products are amplified using PCR. DNAm is evaluated as a value between 0–1, otherwise known as beta values, calculated from the intensity of the methylated and the unmethylated alleles^34^. All samples were assayed by random assignment across a single 96 well plate with parallel processing. It is important to note that our analysis did not differentiate between DNAm and hydroxymethylation. DNA hydroxymethylation has been hypothesized to have a functional role outside of DNA demethylation, but as it is present at significant levels only in neural and pluripotent cells, this function is not likely to be important in non-neural tissue^51^.

### Data Quality Control and Preprocessing

Before analysis, data was subjected to stringent quality control. First, we removed all probes for SNPs and X-chromosome sites. Next, CpG sites where >23 subjects had beta values of zero, as well as sites where >3 subjects had signals below background detection were removed. Lastly, we removed probes with polymorphic CpGs that may interfere with the analysis^14^,^27^. This left 998 CpG loci, and 25 subjects ranging from 26 to 45 years in age (10 male and 15 female). Methylation measurements of zero were replaced with the minimum measurement for either PBMC (0.033) or BEC (0.03). All analysis was performed using the R statistical computing software (http://www.r-project.org), and statistical tests were conducted with alpha level of 0.05.

### Differential and Tissue Specific DNAm

A probe-wise paired two-sample t-test (n = 25, two tailed) not assuming equal variance was used to compare the equality of mean probe methylation across the two tissues. False discovery rate was adjusted using Benjamini Hochberg correction, after which adjusted p-value = 0.001 was used as the significance level. Mean beta values were obtained for the 998 probes in each tissue by averaging across the 25 individuals. Cross-tissue Δβ difference was calculated on a probe-wise basis by calculating the numerical difference between the average beta values. All statistical tests were conducted with alpha level of 0.05 (the probability of rejecting a true null hypothesis), which determines the probability of making a type I error.

Probes with significant differences in beta values between the two tissues were identified using a combination of t-test results and Δβ values. Tissue specific probes were defined as those with t-test p-value smaller than 0.001 and having an absolute Δβ of larger than 0.2. Highly tissue specific probes are defined as those with t-test p-value smaller than 0.001 and absolute Δβ larger than 0.4.

### Intraclass Correlation

The 998 probes were separated into three categories: 1. High CpG density (HC, CG content >55%, Obs/Exp CpG ratio >0.75 and length >500 bps) probes; 2. Intermediate CpG density (IC, CG content >50%, Obs/Exp CpG ratio >0.48 and length >200 bps) probes; and 3. Low CpG density (LC, non-islands or low-density CpG regions, non-HC/IC regions) probes^26^. Probe-wise Intraclass correlation coefficient (ICC) was calculated using the one-way consistency model in the irr R package. Calculation of ICC was done as described in equation (1). For probe *i*,
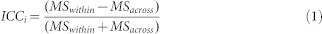
Where MS_within_ is the mean square value for within tissue difference, and MS_across_ is the mean square value for across tissue difference. As ICC_i_ approaches +1, the within tissue difference is larger than the between-tissue difference for probe *i*; and as ICC_i_ approaches −1, the between-tissue difference is larger than the within-tissue difference. Results from ICC offered insight into CpG regions that were more likely to display methylation difference across tissues.

### Correlation between tissues

Pearson's two-tailed pairwise correlation was used to assess the comparability of inter-individual DNAm levels across tissues. A Pearson's correlation coefficient was calculated for each CpG site by correlating their methylation measurements for each of the 25 individuals in BEC to their measurements in PBMC.

### Concordance in probe variance across tissues

DNAm range was visualized on a probewise basis using mean normalization. Range values were calculated as described in equation (2). For individual j of probe i, 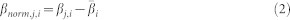
Due to truncation of variance at the extremes of DNAm, a beta value transformation was applied^13^,^52^,^53^. The transformation was achieved as described in equation (3). For individual j of probe i,
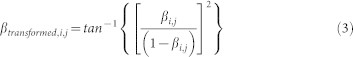
After transformation, variance was also calculated for each probe and SD was derived by taking the square root value of variance. A levene's test for equality of variance was conducted using the lawstat package in R using transformed BEC and PBMC DNAm values, and tissue as the grouping factor. P-values from the test were adjusted for false discovery rate using Benjamini Hochberg correction, and an adjust p-value of 0.05 was used as the cut-off for significance.

### DAVID Functional Enrichment Analysis

Entrez gene-ID corresponding to the 998 CpG sites involved in the analysis was used as background for DAVID GO analysis^37^. CpG probe lists were tested for enrichment of GO subcategories cellular component and biological process. Functional enrichment scores of >1.3 were defined as significantly enriched.

### DNAm and demographics

Association between DNAm and demographic factors gender (15 male vs 10 female) and race (18 Caucasian versus 7 non-Caucasian) was investigated using the two-tailed Wilcoxon rank sum test for categorical variables. Multiple testing adjustments were done using the Storey's q value method in the qvalue Bioconductor package. A q value of 0.05 was defined as the significance threshold. For risk factors BMI and alcohol consumption, association with DNAm was tested using a two-tailed Pearson's correlation. Multiple testing adjustments were again carried out using the q value method, and probes with significant association to risk factors were defined as those with q value <0.05.

## Author Contributions

E.C., S.M.N., H.B.F. and G.E.M. participated in data acquisition, while R.J. and M.J.J. were responsible for conception and design of data analysis. R.J., M.J.J., as well as M.S.K. carried on subsequent analysis examination, results interpretation, and manuscript drafting. Finally, everyone on the author list was responsible for content revision and final approval.

## Supplementary Material

Supplementary Information Supplementary Information

## Notes

The dataset investigated in this study has been deposited in the Gene Expression Omnibus repository with the accession number GSE53396.
