# Supplementary material for: Discordance of DNA Methylation Variance Between two Accessible Human Tissues
Source: Sci Rep. 2015 Feb 9;5:8257. doi: 10.1038/srep08257 (PMC4321176; doi:10.1038/srep08257)
Supplement: Supplementary Information [file srep08257-s1.doc]

**Subject Area:**

Human DNA methylation

**Correspondence and requests for materials:**

[msk@cmmt.ubc.ca](mailto:msk@cmmt.ubc.ca)

**Title:**

Discordance of DNA Methylation Variance Between two Accessible Human Tissues

Ruiwei Jiang^1^*, Meaghan J. Jones^1^*, Edith Chen^2^, Sarah M. Neumann^1^, Hunter B. Fraser^3^, Gregory E. Miller^2^†, and Michael S. Kobor^1,4^†

^1^Centre for Molecular Medicine and Therapeutics, Child and Family Research Institute, Department of Medical Genetics, University of British Columbia, Vancouver, BC, V5Z 4H4, Canada.

^2^ Department of Psychology and Institute for Policy Research, Northwestern University, Evanston, IL 60208, United States.

^3^ Department of Biology, Stanford University, Stanford, CA 94305, United States

^4^Human Early Learning Partnership, School of Population and Public Health, University of British Columbia, Vancouver, BC, V6T 1Z8, Canada.

* These authors contributed equally to this work

†Co-corresponding authors

**
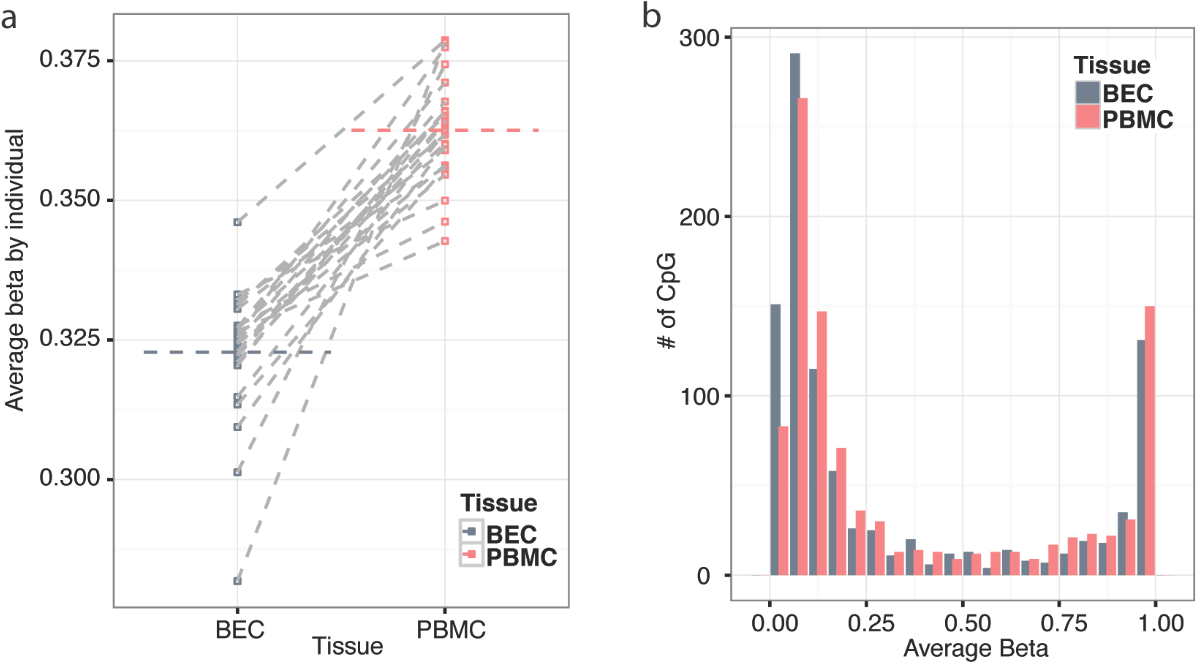
**

**Figure S1.** BEC displayed overall lower DNAm than PBMC (A) Sample mean DNAm levels for BEC (grey) and PBMC (red). Dashed red line represents mean DNAm for each tissue. Each of the 25 individuals displayed higher average PBMC DNAm than BEC DNAm. (B) Distribution of PBMC (grey) and BEC (red) beta values. BEC had more hypomethylated CpG sites than PBMC, and PBMC had slightly more hypermethylated sites than BEC.

**
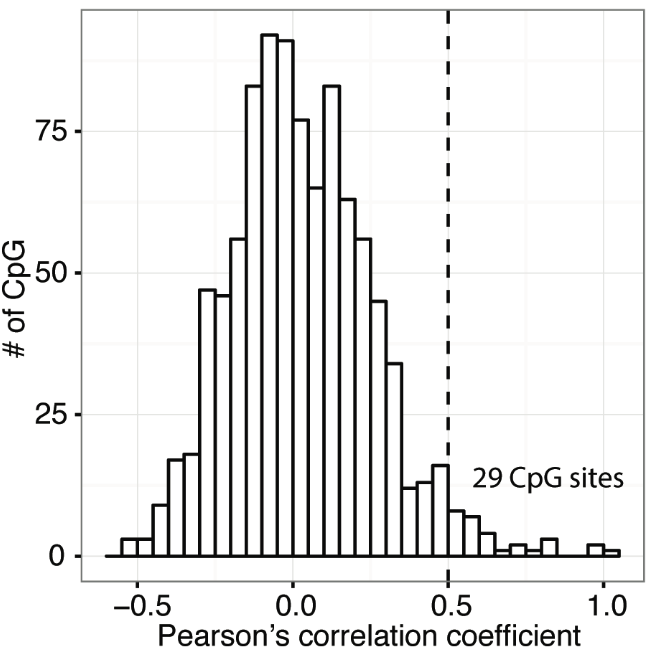
**

**Figure S2**. Relative levels of DNAm among individuals were not translatable across tissues. Distribution of Pearson’s correlation coefficient (r) for probe-wise correlation between BEC and PBMC. Dashed line represent Pearson’s correlation coefficients of 0.5.

**Table S1**. Demographic information for the 25 healthy subjects involved in the study.

| Subject ID | Age | Gender | Race | BMI | Alcohol drinks per week |
| --- | --- | --- | --- | --- | --- |
| 857 | 27 | 1 | 1 | 26.66 | 20 |
| 858 | 35 | 2 | 1 | 22.3 | 1 |
| 859 | 30 | 1 | 1 | missing | 2.25 |
| 860 | 31 | 2 | 0 | 24.41 | 0 |
| 861 | 35 | 1 | 1 | 24.89 | 10 |
| 862 | 33 | 2 | 1 | 21.46 | 0 |
| 864 | 38 | 1 | 1 | 24.4 | 5 |
| 866 | 39 | 2 | 1 | 18.18 | 2.5 |
| 867 | 40 | 2 | 0 | 19.7 | 0 |
| 868 | 36 | 1 | 1 | 24.94 | 12 |
| 869 | 32 | 2 | 1 | 23.76 | 4 |
| 870 | 27 | 2 | 0 | 47.59 | 0 |
| 871 | 45 | 2 | 0 | 22.83 | 0 |
| 872 | 26 | 1 | 1 | 20.64 | 9 |
| 874 | 42 | 2 | 0 | 19.71 | 0 |
| 875 | 26 | 2 | 0 | 25.08 | 8 |
| 876 | 37 | 2 | 1 | 23.76 | 1.25 |
| 877 | 41 | 2 | 1 | 21.02 | 2 |
| 881 | 39 | 2 | 1 | 21.84 | 0 |
| 882 | 27 | 1 | 1 | 22.09 | 0 |
| 884 | 39 | 1 | 1 | 29.89 | 15.5 |
| 885 | 39 | 1 | 1 | 24.81 | 0 |
| 887 | 29 | 2 | 1 | 33.72 | 0 |
| 889 | 29 | 2 | 0 | 23.43 | 0 |
| 890 | 27 | 1 | 1 | 21.87 | 3 |

**Table S2**. DAVID gene ontology enrichment for tissue-specific probes using sub-ontologies biological process and cellular component.

| **Cluster** | **Enrichment score** | **GO term** | **# of associated loci** |
| --- | --- | --- | --- |
| 1 | 1.63 | Extracellular region (GO:0005576) | 47 |
| 2 | 1.59 | Immune system process (GO:0002376) | 29 |
|  |  | Cytokine activity (GO:0005125) | 11 |

**Table S3.** Probes in PBMC found to display high confidence associations with gender and BMI (FDR≤0.05).

| **Gender associated high confidence probes (40)** | | | | | |
| --- | --- | --- | --- | --- | --- |
|  | **Probe ID** | **CpG Island** | **Gene Symbol** | **Chromosome** | **CpG Density** |
| 1 | ABO_P312_F | Y | ABO | 9 | HCP |
| 2 | ADCYAP1_P398_F | Y | ADCYAP1 | 18 | HCP |
| 3 | APBA1_P644_F | Y | APBA1 | 9 | HCP |
| 4 | COL4A3_E205_R | Y | COL4A3 | 2 | HCP |
| 5 | CYP1B1_P212_F | Y | CYP1B1 | 2 | HCP |
| 6 | DLC1_P695_F | N | DLC1 | 8 | LCP |
| 7 | DST_E31_F | Y | DST | 6 | HCP |
| 8 | ETS1_E253_R | Y | ETS1 | 11 | HCP |
| 9 | HOXA5_P479_F | Y | HOXA5 | 7 | HCP |
| 10 | HPSE_P29_F | Y | HPSE | 4 | HCP |
| 11 | IGF1_E394_F | N | IGF1 | 12 | LCP |
| 12 | IGFBP7_P371_F | Y | IGFBP7 | 4 | HCP |
| 13 | IHH_P529_F | Y | IHH | 2 | HCP |
| 14 | IL16_P93_R | N | IL16 | 15 | LCP |
| 15 | IL2_P607_R | N | IL2 | 4 | LCP |
| 16 | JAG2_P264_F | Y | JAG2 | 14 | HCP |
| 17 | MAP3K9_E17_R | Y | MAP3K9 | 14 | HCP |
| 18 | MLH1_P381_F | Y | MLH1 | 3 | HCP |
| 19 | MLH3_P25_F | Y | MLH3 | 14 | HCP |
| 20 | MMP10_E136_R | N | MMP10 | 11 | LCP |
| 21 | MPO_P883_R | N | MPO | 17 | LCP |
| 22 | MSH3_E3_F | Y | MSH3 | 5 | HCP |
| 23 | PDE1B_P263_R | Y | PDE1B | 12 | ICP |
| 24 | PGF_E33_F | Y | PGF | 14 | HCP |
| 25 | PLAGL1_P236_R | Y | PLAGL1 | 6 | HCP |
| 26 | PTGS1_P2_F | Y | PTGS1 | 9 | ICP |
| 27 | RASA1_E107_F | Y | RASA1 | 5 | HCP |
| 28 | SERPINA5_E69_F | N | SERPINA5 | 14 | LCP |
| 29 | SFTPB_P689_R | N | SFTPB | 2 | LCP |
| 30 | SOX1_P1018_R | Y | SOX1 | 13 | HCP |
| 31 | STK11_P295_R | Y | STK11 | 19 | HCP |
| 32 | TCF7L2_P193_R | Y | TCF7L2 | 10 | HCP |
| 33 | TDGF1_P428_R | N | TDGF1 | 3 | ICP |
| 34 | TFDP1_P543_R | Y | TFDP1 | 13 | HCP |
| 35 | TMEFF1_E180_R | Y | TMEFF1 | 9 | HCP |
| 36 | TP73_E155_F | Y | TP73 | 1 | HCP |
| 37 | TP73_P496_F | Y | TP73 | 1 | HCP |
| 38 | TRAF4_P372_F | Y | TRAF4 | 17 | HCP |
| 39 | WT1_E32_F | Y | WT1 | 11 | HCP |
| 40 | XPC_P226_R | Y | XPC | 3 | HCP |
| **BMI associated high confidence probes (51)** | | | | | |
|  | **Probe ID** | **CpG Island** | **Gene Symbol** | **Chromosome** | **CpG Density** |
| 1 | AATK_P519_R | Y | AATK | 17 |  |
| 2 | ABCA1_P45_F | Y | ABCA1 | 9 | HCP |
| 3 | ABO_E110_F | Y | ABO | 9 | HCP |
| 4 | ACVR1_P983_F | N | ACVR1 | 2 | HCP |
| 5 | ACVR1C_P115_R | Y | ACVR1C | 2 | HCP |
| 6 | AREG_E25_F | Y | AREG | 4 | HCP |
| 7 | CASP2_P192_F | Y | CASP2 | 7 | HCP |
| 8 | CCND2_P898_R | Y | CCND2 | 12 | ICP |
| 9 | CD81_P272_R | Y | CD81 | 11 | HCP |
| 10 | CDKN2B_E220_F | Y | CDKN2B | 9 | HCP |
| 11 | COL18A1_P365_R | Y | COL18A1 | 21 | LCP |
| 12 | COL4A3_P545_F | Y | COL4A3 | 2 | HCP |
| 13 | CPNE1_P138_F | Y | CPNE1 | 20 | - |
| 14 | EPHB6_E342_F | Y | EPHB6 | 7 | HCP |
| 15 | EVI2A_P94_R | N | EVI2A | 17 | LCP |
| 16 | FGFR1_E317_F | Y | FGFR1 | 8 | HCP |
| 17 | FHIT_E19_R | Y | FHIT | 3 | ICP |
| 18 | HDAC7A_P344_F | N | HDAC7A | 12 | - |
| 19 | HHIP_E94_F | Y | HHIP | 4 | HCP |
| 20 | HLA-DPB1_E2_R | N | HLA-DPB1 | 6 | LCP |
| 21 | HOXA5_E187_F | Y | HOXA5 | 7 | HCP |
| 22 | HOXB2_P488_R | N | HOXB2 | 17 | ICP |
| 23 | IGSF4C_P533_R | Y | IGSF4C | 19 | HCP |
| 24 | IHH_P529_F | Y | IHH | 2 | HCP |
| 25 | INHA_P1144_R | Y | INHA | 2 | LCP |
| 26 | IRAK3_P13_F | Y | IRAK3 | 12 | HCP |
| 27 | ITGA6_P718_R | Y | ITGA6 | 2 | - |
| 28 | ITGB4_E144_F | Y | ITGB4 | 17 | HCP |
| 29 | MAGEL2_P170_R | Y | MAGEL2 | 15 | ICP |
| 30 | MAP3K8_P1036_F | Y | MAP3K8 | 10 | HCP |
| 31 | MAPK14_P327_R | Y | MAPK14 | 6 | HCP |
| 32 | MAPK9_P1175_F | N | MAPK9 | 5 | LCP |
| 33 | MCM2_P241_R | Y | MCM2 | 3 | HCP |
| 34 | MOS_P27_R | Y | MOS | 8 | ICP |
| 35 | MST1R_E42_R | Y | MST1R | 3 | HCP |
| 36 | MUC1_P191_F | Y | MUC1 | 1 | ICP |
| 37 | MYOD1_P50_F | Y | MYOD1 | 11 | HCP |
| 38 | NBL1_P24_F | N | NBL1 | 1 | LCP |
| 39 | NPR2_P1093_F | Y | NPR2 | 9 | HCP |
| 40 | NTRK2_P10_F | Y | NTRK2 | 9 | HCP |
| 41 | NTSR1_E109_F | Y | NTSR1 | 20 | HCP |
| 42 | PRSS8_E134_R | Y | PRSS8 | 16 | LCP |
| 43 | PTGS1_P2_F | Y | PTGS1 | 9 | ICP |
| 44 | PTK2_P735_R | Y | PTK2 | 8 | HCP |
| 45 | PURA_P928_R | Y | PURA | 5 | HCP |
| 46 | SKI_E465_R | Y | SKI | 1 | HCP |
| 47 | SMARCA3_P109_R | Y | SMARCA3 | 3 | HCP |
| 48 | SNRPN_E14_F | N | SNRPN | 15 | ICP |
| 49 | TFAP2C_P765_F | Y | TFAP2C | 20 | HCP |
| 50 | TMPRSS4_E83_F | N | TMPRSS4 | 11 | LCP |
| 51 | ZIM2_E110_F | Y | ZIM2 | 19 | - |
